# Effect of data normalization on fuzzy clustering of DNA microarray data

**DOI:** 10.1186/1471-2105-7-134

**Published:** 2006-03-14

**Authors:** Seo Young Kim, Jae Won Lee, Jong Sung Bae

**Affiliations:** 1 Research Institute for Basic Science, Chonnam National University, Gwangju, 500-757, Korea; 2 Department of Statistics, Korea University, Seoul, Korea; 3 Department of Statistics, Chonnam National University, Gwangju, 500-757, Korea

## Abstract

**Background:**

Microarray technology has made it possible to simultaneously measure the expression levels of large numbers of genes in a short time. Gene expression data is information rich; however, extensive data mining is required to identify the patterns that characterize the underlying mechanisms of action. Clustering is an important tool for finding groups of genes with similar expression patterns in microarray data analysis. However, hard clustering methods, which assign each gene exactly to one cluster, are poorly suited to the analysis of microarray datasets because in such datasets the clusters of genes frequently overlap.

**Results:**

In this study we applied the fuzzy partitional clustering method known as Fuzzy C-Means (FCM) to overcome the limitations of hard clustering. To identify the effect of data normalization, we used three normalization methods, the two common scale and location transformations and Lowess normalization methods, to normalize three microarray datasets and three simulated datasets. First we determined the optimal parameters for FCM clustering. We found that the optimal fuzzification parameter in the FCM analysis of a microarray dataset depended on the normalization method applied to the dataset during preprocessing. We additionally evaluated the effect of normalization of noisy datasets on the results obtained when hard clustering or FCM clustering was applied to those datasets. The effects of normalization were evaluated using both simulated datasets and microarray datasets. A comparative analysis showed that the clustering results depended on the normalization method used and the noisiness of the data. In particular, the selection of the fuzzification parameter value for the FCM method was sensitive to the normalization method used for datasets with large variations across samples.

**Conclusion:**

Lowess normalization is more robust for clustering of genes from general microarray data than the two common scale and location adjustment methods when samples have varying expression patterns or are noisy. In particular, the FCM method slightly outperformed the hard clustering methods when the expression patterns of genes overlapped and was advantageous in finding co-regulated genes. Thus, the FCM approach offers a convenient method for finding subsets of genes that are strongly associated to a given cluster.

## Background

DNA microarray technology has the potential to create enormous quantities of data in short times. The vast amounts of information generated by microarray experiments have led to the need for methods for analyzing such data. Clustering has proved to be an important tool for this purpose. The ability of clustering methods to extract groups of genes with similar functions from huge datasets stems from the fact that genes with similar functions evince similar expression patterns of co-regulation [1,2].

Clustering methods can be broadly classified into two types according to the method adopted to define clusters [3]: hierarchical and partitional clustering methods. Hierarchical clustering [2,4] produces dendrograms, in which each branch represents a group of genes that have a higher order relationship. One major shortcoming of this approach is that it cannot identify co-expressed genes in large gene expression datasets when such datasets are collected under varying conditions [5]. In addition, hierarchical clustering does not produce a unique dendrogram and does not reflect the multiple ways in which expression patterns of genes can be similar [6]. Partitional clustering attempts to directly decompose the dataset into a set of disjoint clusters. The representative Partitioning Around Medoids (PAM) [7], K-means [8], and hierarchical clustering methods assign each gene to a single cluster, even if the expression profile of that gene has a number of similar cluster patterns. Although these methods work well when applied to datasets with well-defined clusters, they are inappropriate for microarray data due to the complicated structures of such biological datasets. In addition, it is difficult to find distinct clusters in gene expression data using hard clustering methods, because clusters of genes in gene expression data do not have well-defined boundaries [2].

To overcome the limitations of these hard clustering methods, here we apply fuzzy partitional clustering based on the Fuzzy C-Means (FCM) algorithm [9,10]. FCM clustering provides a systematic and unbiased way to change precise values into several descriptors of cluster memberships [9]. Thus, this method provides more information regarding the degrees of membership of each gene to each cluster of genes. The main advantage of using fuzzy clustering to analyze gene expression data lies in its ability to handle noisy data [3]. FCM clustering also attempts to find the most characteristic data point in each cluster, which can be considered the center of the cluster, and then the degree of membership for each gene in the cluster [3]. When implementing fuzzy algorithms, it is very important to choose appropriate values for parameters like the weighting exponent, *m *(the so-called fuzzification). Especially, in fuzzy models the minimization criterion for the objective function depends on *m*. In the fuzzy clustering literature, a value of *m *= 2 is commonly used, but this value is not appropriate for gene expression data [11]. Moreover, the optimal value of *m *depends on the dataset used, and the clustering results are sensitive to *m*. Thus the appropriateness of a value of *m *for use in cluster analysis of a dataset is very sensitive to the characteristics of the dataset. Two factors that affect the dataset characteristics are the noisiness of the data and the method used to normalize the dataset. Therefore research into normalizing and removing noise from datasets has been a very important component of previous work on clustering analysis. In particular, for the same dataset and cluster analysis method, we expect that the results of clustering will vary depending on the method used to normalize the data. Normalization of microarray data is required to remove systematic variations introduced in the experiments, which affect the measured expression levels. In microarray experiments, there are many sources of systematic variation, for example differences in labeling efficiency between different fluorescent dyes. We used the two common scale and location transformation methods (we called Z- and R-methods) and Lowess normalization method.

In the present study, we compare FCM clustering with three hard clustering approaches: the PAM, K-means and hierarchical methods. In addition, we evaluate the effect of the method used to normalize microarray data on the results obtained when clustering analysis is applied to the normalized dataset. This comparative analysis is performed using three normalization methods, applied to three microarray datasets and three simulated datasets. In the Materials and Methods section, we introduce the FCM clustering algorithm for finding groups of genes with similar expression patterns in microarray data, and review in detail validation techniques for evaluating the final clusters.

## Results and discussion

Before applying the clustering algorithm to each dataset, all data were preprocessed by appropriate transformation and normalization methods, and determined the optimal fuzzification, *m*. We then used this value of *m *in applying the FCM method to each normalized dataset, and compared the clustering results to determine the effects of the data normalization. After determining the fuzzification, *m*, the number of clusters has to be determined. In the present work, the number of clusters, *c*, was taken as the number that gave the maximum value of the silhouette index. This procedure was performed on three microarray datasets, referred to as the serum [12,13], sporulation [14] and yeast [15] datasets. Finally, we compared the FCM clustering method with other clustering methods using the adjusted Rand index.

### Determination of the fuzzification parameter

**Figure 1:**
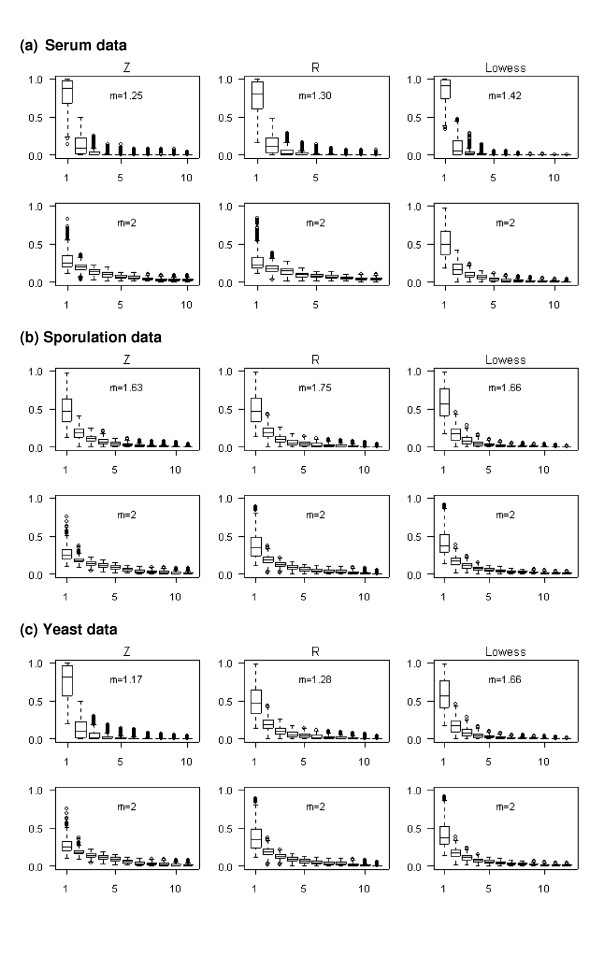
**Boxplots of sorted membership values for real data**. Boxplots of sorted membership values for three real datasets normalized by the Z, R or Lowess method, after FCM clustering using an *m *value of 2 or the optimal value. The *x*-axis indicates the sorted membership values for each gene (highest, second highest, etc.) and the *y*-axis indicates the membership value. (a) serum data, (b) sporulation data and (c) yeast data.

**Figure 2:**
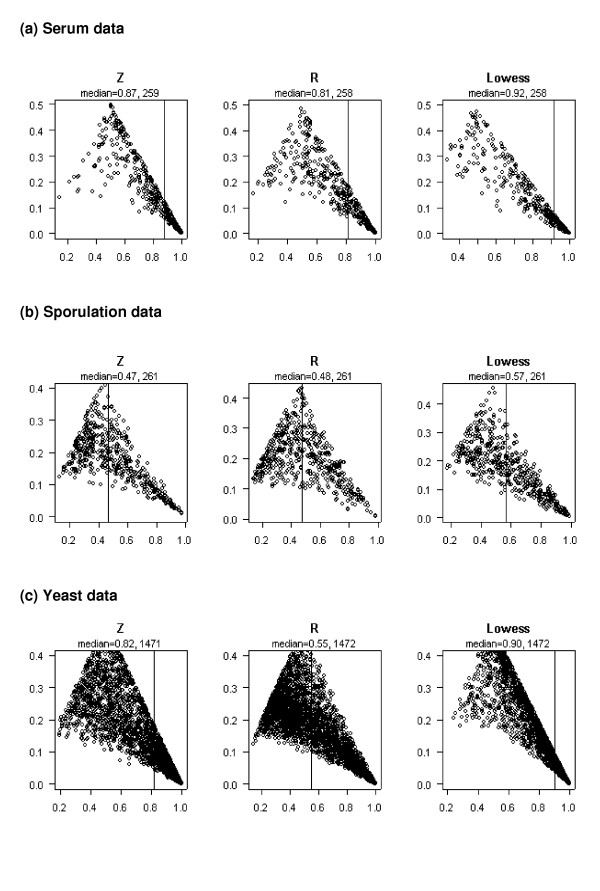
**Scatterplot of the two highest membership values of all genes**. Scatter plots of the two highest membership values of all genes for the optimal *m *in the dataset. The distribution of the highest two membership values is observed using the scatter plot of the first two highest memberships for each gene for the optimal *m *and three normalization methods. The *x *axis indicates the first highest membership, the *y *axis indicates the second highest membership, and the vertical line indicates the median value of the highest membership value. (a) Serum data (b) sporulation data (c) yeast data.

The fuzzification parameter, *m*, was obtained using an approach based on the work of Dembele and Kastner [11]. They proposed that the commonly used value of *m *= 2 is inappropriate for FCM clustering of microarray data. Following this previous work [11], we determined the fuzzification parameter for each normalized dataset. The values of *m *and the number of clusters, *c*, used for the FCM clustering of the three datasets and three normalization methods are given in Table 1. The fuzzification parameter varies depending on both the normalization method and the dataset used. Figure 1 shows boxplots of the membership values for each gene from the FCM clustering of the three datasets after normalization by the three methods. When *m *is fixed to 2, for the sporulation and yeast datasets, the membership values for most genes are close to 1/*c*, and the membership values are relatively insensitive to the normalization method. When the optimal value of *m *is used, by contrast, most membership is shared by the first and second highest membership values. In particular, the first and second highest membership values of all genes are well separated by the Lowess normalization for most of the datasets normalized using this method. Here, the first highest membership values are used to allocate each gene to a single cluster, as in hard clustering methods. In addition, the membership values can be used to find genes strongly associated to given cluster. We used the median of the membership values as the threshold membership for specifying that a gene is tightly associated with a particular cluster [11]. Compared to the only scale and location transformations, normalization using the Lowess method gives many more genes strongly associated to a given cluster due to the high median of the first highest membership in the Lowess method of figure 1 and figure 2.

The distribution of the highest two membership values is observed using a scatter plot of these values for each gene for the optimal *m *by each normalization method (Figure 2). The results displayed in Figures 1 and 2 show that when the optimal *m *is used, the distribution of the membership values varies markedly depending on the normalization method applied to the dataset. In each of the three datasets, the median of the first highest membership differs greatly depending on the normalization method used. For the serum, sporulation and yeast datasets, the median of the first highest membership is greatest when the dataset was normalized using the Lowess method, with values of 0.92, 0.57, and 0.90 respectively. Moreover, in the case of the serum and yeast datasets, the gene activities can practically be determined from their first and second highest membership values, whereas the sporulation dataset has low membership values. On the other hand, the serum dataset shows a larger dispersion of genes over samples than the other two datasets. In addition, for each dataset, the distribution of membership depends on the normalization method applied to the dataset. In the case of the Lowess method, the association of the first and second highest membership is strong in each dataset because the median memberships are relatively high. In the case of the R-transformation, by contrast, only a weak association is observed in each dataset of Figure 2. These results indicate that relatively high membership values can be obtained for the dataset with small variability among samples, and that the Lowess normalization appears to be more effective to the other methods.

### Effect of data normalization

#### Sensitivity of clustering to the fuzzification parameter

**Figure 3:**
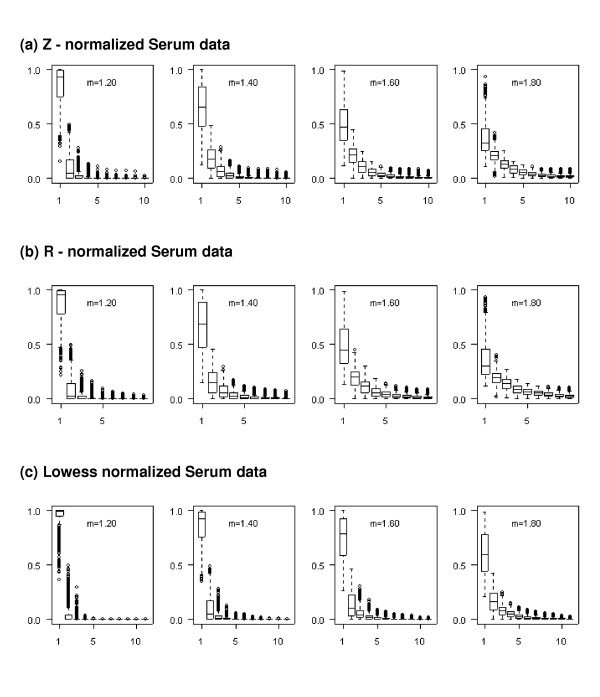
**Boxplots of sorted membership values for the serum dataset for four different *m *values**. Boxplots of sorted membership values for the serum dataset, for three normalization methods and four values of *m *ranging from 1.2 to 1.8. The *x *axis indicates the number of clusters, and the *y *axis indicates membership value (a) Z-normalization (b) R-normalization (c) Lowess-normalization.

Box plot representations of the membership values for each gene, in decreasing order, are shown in Figure 3 for four values of *m *(1.2, 1.4, 1.6, and 1.8). These values were selected based on the knowledge that if *m *is close to 1, the clusters obtained are almost crisp, and if *m *is close to 2, the distribution of membership values becomes more spread out, with *m *values higher than 2 resulting in the loss of any useful information about membership values [5]. To identify the effect of normalization technique on the clustering results, we applied each normalization method to the serum dataset and compared the membership values obtained by the FCM method for a range of *m *values. This dataset was chosen because it has a large variation of gene expression levels, unlike the other two datasets. For the data transformed by the Z and R methods, the first highest membership of genes decreases rapidly with increasing *m*. In contrast, the data normalized using the Lowess method shows the highest membership values of all genes and the membership values decrease slowly with increasing *m*. The data in Figure 3 thus indicate that the clusters obtained using Lowess-normalized data are more robust to different values of *m *than those obtained using only Z- and R-transformed data.

#### Stability of clusters using FCM

**Figure 4:**
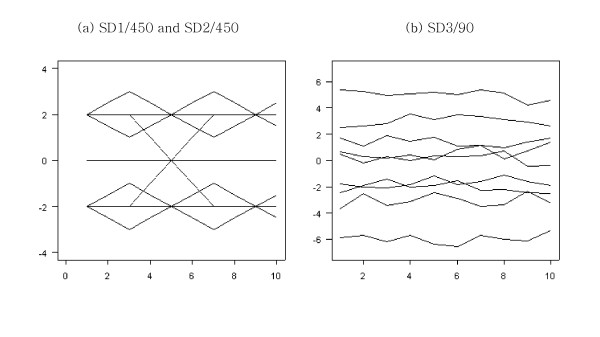
**Expression patterns of the simulated datasets**. (a) SD1/450 and SD2/450 have nine distinct patterns over 10 time points, and each cluster contains 50 genes; (b) SD3/90 contains nine overlapped patterns over 10 time points, and each cluster contains 10 genes.

We compared the performances of the three normalization methods when applied to three simulated datasets, referred to as SD1/450 and SD2/450 and SD3/90. The true number of clusters in each of these datasets was known to be *c *= 9; the nine clusters in SD1/450 and SD2/450 are well separated, whereas those in SD3/90 have some overlap as shown in Figure 4. Here the performance of each normalization method was quantified using the adjusted Rand index, which is a measure of the agreement of determined clusters in comparison with the true clusters. Cluster allocations were determined from the first highest membership, and compared with the correct clusters for FCM clustering. The results of these comparisons are shown in Table 2. The values of the adjusted Rand index obtained by comparing the true clusters to those obtained by applying FCM clustering (using both the optimal *m *value and *m *= 2) to the datasets normalized by the Z, R and Lowess methods showed that the Lowess-normalized dataset performed better than those normalized by the other two only scale and location transformation methods. In fact, even when *m *was fixed at 2, the analysis results obtained using the Lowess normalized data were similar to those obtained using the optimal *m*. In contrast, the Z and R transformations did not work well.

The results presented above (Table 2 and Figure 1) showed that the Lowess normalization outperformed the other two transformations methods. To further analyze the efficacy of the FCM approach, we used the adjusted Rand index to compare the true clusters in the SD1/450, SD2/450, and SD3/90 datasets with those obtained by applying FCM using the optimal *m *values for each dataset and three hard clustering methods (PAM, K-means, and Hierarchical clustering method) to these three simulated datasets after Lowess normalization. The results are shown in Table 3. For the SD1/450 and SD2/450 datasets, which have well separated clusters, the PAM and Hierarchical clustering methods performed very well; the performance of the FCM clustering was good, although not as good as those of the PAM and Hierarchical clustering methods. In the case of the SD3/90 dataset, which has overlapping expression patterns across samples, FCM clustering slightly outperform other hard clustering methods. Also, FCM clustering has similar values of index over three datasets, in contrast, hard clusterings have very different values of index. This means hard clustering is unstable on data with overlapping clusters contrary to FCM clustering. FCM clustering of microarray data using the common fuzzification parameter value of *m *= 2 is known to give poor performance compared to the results of hard clustering; however, the present results show that if the optimal fuzzification parameter value is used, the clustering performance similar to that obtained using hard clustering is achieved for the dataset with overlapping clusters.

On the basis of these results, we can find merits for FCM clustering as follows; first, the membership values of FCM clustering can be used on several levels. Basically, the membership values can be used assign each gene to one cluster, resulting in an effect equivalent to the hard clustering method; second, using membership values, it is possible to find genes which display a strong association to a given cluster and the most likely to work together even in different pathways. For example, there are 258 strong association genes in serum dataset as shown in figure 2(a). In the figure, we find genes with first highest membership values that are greater than the median value of 0.87. Thus we can expect these genes are always co-expressed and co-regulated under all conditions.

### Comparative analysis of FCM and PAM clustering of noisy data

The FCM method provides a systematic, unbiased formalism for transforming precise values into several descriptors of cluster membership [9]. Compared to the hard clustering methods, the FCM method provides more information on the degree of biological similarity of each gene. The main advantage of the FCM method in microarray data analysis is that it explains the noise in the data. Expression levels with low membership values for all clusters may be considered as noise points, and the final clustering results may depend on those noise points. In other words, because noisy data contain many points that have low membership values for all clusters, such data cannot be well clustered across all samples. Therefore, identifying noise points is essential to gene clustering using the FCM method.

**Figure 5:**
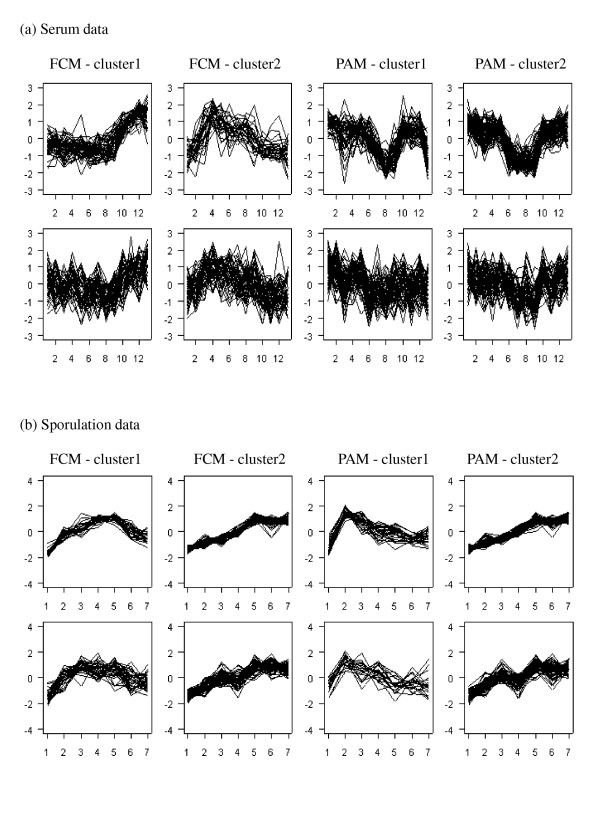
**Identification of influence of noise by FCM and PAM clustering**. Expression patterns for any two clusters between original and noisy data clustering for FCM and PAM clustering in serum and sporulation datasets. For each dataset, upside line of figure indicates the original clustering and the downside line indicates the noisy data clustering, and first and second columns are the results of FCM clustering and others two are the results of PAM clustering. The *x *axis indicates the number of clusters, and *y *axis indicates the values of data points (a) Serum data (b) sporulation data.

**Figure 6:**
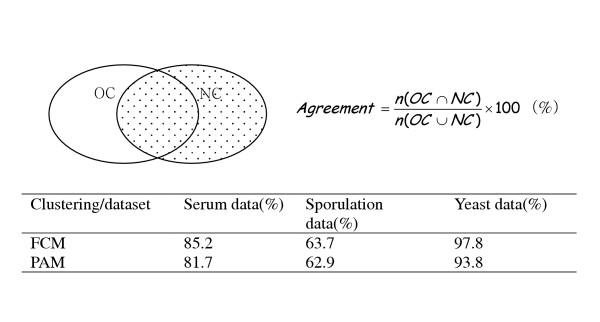
**Calculation of agreement between original and noisy data clustering**. OC indicates original data clustering of genes, and NC indicates noisy data clustering of genes. *n*(*OC *∩ *NC*) is the number of genes assigned to the same clusters in both clusterings, and *n*(*OC *∪ *NC*) is the number of genes assigned to one cluster at least for both clusterings. Here, the agreement computes the degree of agreement between the original and noisy datasets, where the noisy dataset was generated by adding a Gaussian random variable of mean 0, and standard deviation 1 to the original dataset.

To test the ability of the FCM method to handle noisy data, we performed additional clustering calculations on versions of the serum, yeast, and sporulation microarray datasets that had been modified to include noise and subsequently normalized by the Lowess method as shown in Figure 5. The noisy datasets were generated by adding a small Gaussian random variable of mean 0 and standard deviation 1 to the gene expression levels in the original microarray dataset, and then the resulting dataset was normalized by the Lowess method. The noisy gene expression datasets were clustered and the results compared to the clustering of the original dataset by measuring the agreement between the clustering results as shown in Figure 6. The agreement measures the degree to which the pairs of genes allocated to a cluster in one clustering calculation agree with those allocated to the same cluster in another calculation. To identify the ability of the FCM method to cluster noisy data, we compared the results obtained using this approach with those obtained using PAM clustering known as representative partitional clustering method with good performance. For each type of dataset, we used the same number of clusters for each clustering method in both the original and noisy datasets.

Finally, to evaluate the robustness of FCM clustering to noise in data, we computed the agreement of pairs of genes assigned to the same clusters in the clusterings of the original dataset and the corresponding noisy dataset as shown in Figure 6. In the analysis of the original serum dataset and its noisy counterpart, PAM and FCM clustering allocated similar percentages (average 81.7% and 85.2% respectively) of the pairs of genes to the same cluster in the analyses of the original and noisy datasets. In the clustering analysis of the original and noisy sporulation and yeast datasets by FCM clustering, however, 63.7% and 97.8% of pairs were in the same clusters, respectively, which were slightly higher than the percentages obtained using PAM clustering (62.9% and 93.8% respectively). These results are briefly illustrated with the two serum and sporulation datasets which indicate the low degree of agreement of pairs of genes assigned to the same clusters between original and noisy added datasets. Figure 5 shows the expression patterns from the original and noisy serum and sporulation datasets for cluster1 and cluster2 obtained using FCM and PAM respectively. The upside and the downside of the figures indicate clustering patterns for original and noisy dataset respectively. FCM clustering of the noisy and original datasets gives similar expression patterns for cluster1, and similar patterns for cluster2. The PAM clustering, in contrast, gives different expression patterns for cluster1 depending on whether the original data or its noisy counterpart is used, and likewise for cluster2. In the clustering analysis of the original and noisy sporulation datasets as shown in Figure 5(b), the FCM and PAM clusterings show similar expression patterns for each cluster from the original dataset and its counterpart from the noisy dataset.

From these results, we can see that the FCM clustering method is slightly superior to the PAM method in extracting expression patterns from datasets with larger variations across samples, such as the serum dataset, whereas the FCM and PAM methods show similar performance when applied to data with smaller variations, such as the sporulation and yeast datasets. Importantly, FCM clustering is a little better than the PAM method at handling noisy datasets. In summary, hard clustering methods perform well in particular applications, and are often used for clustering microarray data. However, these methods cannot find co-expressed genes under all conditions and in all samples and do not reflect the multiple ways in which the expression patterns of genes can be similar when analyzing large amounts of noisy microarray data collected under various biological conditions.

## Conclusion

Microarray technology enables the simultaneous measurement of expression levels of thousands of genes. However, the vast amounts of data generated in microarray experiments have led to the need for methods for analyzing such data. Clustering has proved to be an important tool for this purpose. The first step in most statistical analyses of microarray data is to normalize the data so as to remove systematic variations due to non-biological factors. However, the results of clustering analysis of a normalized dataset may depend on the normalization procedure used. In the present study, we considered the effect of data normalization on the results obtained when clustering analysis was applied to the normalized dataset. Six datasets were used: three microarray datasets and three simulated datasets. In particular, we concentrated on the sensitivity of FCM clustering analysis to data normalization and to the value of the fuzzification parameter. To elucidate the performance of FCM clustering relative to other methods, we compared the FCM clustering results with those obtained by applying hard clustering methods to the same normalized datasets. In these comparisons, the performance of each method was quantified using the adjusted Rand index. We found that for all of the datasets examined, Lowess normalization of the dataset gave superior cluster robustness and accuracy compared to the two common data transformations when FCM clustering was applied to the normalized dataset. Moreover, the Lowess normalization method gave robust clustering results when applied to noisy datasets and to datasets containing overlapping clusters.

FCM clustering is a convenient method to find genes exhibiting strong associations to given clusters. Besides, sub-groups of genes can be expected to be contained in several pathways and thus assigned to several clusters but still be co-acting under all conditions. Especially, the performance of FCM clustering is similar to that of hard clustering in respect of allocating a gene to a single cluster for noisy data. The hard clustering forcedly assigns all genes to a respective cluster, even those for which the variations in expression do not fit into any global pattern [11]. In FCM clustering, the genes can belong to more than one cluster where the genes may only be marginally relevant for biological significance of the cluster [11]. Therefore, this method is very useful if we focus on finding genes showing coherent behaviour within clusters. We also expect the fuzzy type clustering might be a significant tool to dissect the several regulatory pathways that control the gene expression patterns of given genes when handling complex datasets.

As the related work, Kim et al. [16] presented the comparative results of three fuzzy type clustering such as FCM, Possibilistic c-means and Fuzzy possibilistic c-means using a common value of 2 as the fuzzification parameter, and Belacel et al. [5] compared performance of Fuzzy J-means and VNS method to that of FCM. They also proposed that the performance of the FCM, without considering effect of data normalization, is slightly weaker than those of other fuzzy methods respectively. However, according to Dembele and Kastner [11], it is not appropriate for fuzzification parameter to be set to a common value of 2 when the fuzzy method is applied to microarray data cluster analysis. Thus, the work of Kim et al. [16] may be clear in only a few cases, but it seems to be difficult to select the best one of three fuzzy methods because the optimal fuzzification parameter was not used. On the other hands, as mentioned earlier, microarray data normalization is basically an important step for obtaining data that are reliable and usable for subsequent analysis. Our analysis also presented that the choice of different normalization methods drastically affects the result of the cluster analysis. In the light of Belacel et al. [5], FCM was inferior to other methods for some dataset but all the three methods had similar performance for the dataset with large variation for each group and for each sample point. Again, the FCM method is faster method compared to the other two methods [5]. Also, the performance of FCM method can be as good as those of Fuzzy J-means or VNS method when the refined data normalization such as the Lowess method is used. Besides, the FCM method can typically be chosen to classify microarray data because a popular and freely available implementation is available in the statistical software package R. Moreover, various other freely available microarray data handling packages have incorporated this FCM method. In summary, we would emphasize that the identification of these two factors that affect cluster results is required when fuzzy clustering methods are used in the microarray data analysis successfully.

Although in the present work the Lowess normalization was found to be superior to the other two normalization methods examined in transforming of data scale and location (Table 2), previous studies have described the problems associated with choosing the parameter values for the Lowess method [17], and Berger et al. [17] pointed out that the normalization results obtained using this method may depend on the parameter values used. In the recent literature on the normalization of microarray data, Zhao et al. [18] presented a mixture model based method and highlighted the importance of normalization in microarray data analysis to find differentially expressed genes across arrays. To our knowledge, however, the present study is the first to test the normalization effect in cluster analysis of microarray data. In future work, we plan to further evaluate normalization methods in regard to microarray experiment conditions, data formats, and the degree of variation in detail. The information gained from such a study should aid in extracting biological information, such as co-expressed genes with similar expression patterns or genes that act in concert in cells, from microarray datasets. For example, this approach could be used to extract biological information that is important for all lung cancer cells regardless of the cell type.

## Methods

### Datasets

#### Simulated data

Three simulated datasets generated around nine distinct temporal patterns over ten time points were considered (Figure 4). The first set (SD1/450) consisted of a total of 450 genes separated into 9 patterns containing 50 genes each. Independent random variables were added to these mean expression-ratio values. Two hundred and twenty five of the total genes were generated from a normal distribution with mean 0 and standard deviation 1, and the remaining 225 were generated from an exponential distribution with location -0.2 and scale 0.2. In the second set (SD2/450), all genes were generated from a normal distribution of mean 0 and standard deviation 1. The first and second datasets were based on the simulated datasets reported in [19] and [20] respectively. The third set (SD3/90) was based on a previously reported simulated dataset [5]. The mean expressions in each of the nine patterns used were -6, -4, -2, -1.5, 0, 0.5, 2, 3, and 5 over different time points, and independent normal variates with mean 0 and standard deviation 1 were added to the nine different mean expression levels.

#### Serum data

The serum dataset used in the present work is described in [12] and [13]. The expression levels of 8613 genes were measured over 24 hours at 12 time points (0, 0.25, 0.15, 1, 2, 4, 6, 8, 12, 16, 20, and 24 hours). We used the 517 genes whose expression varied in response to serum concentration in human fibroblasts.

#### Sporulation data

We used the previously reported gene expression dataset recorded during yeast sporulation [14], which is publicly available [21]. This dataset consists of 6118 genes in the yeast genome measured at seven time points (0, 0.5, 2, 5, 7, 9 and 11.5 hours) during the sporulation process. We used 513 genes that were found to be significantly upregulated during the process [14].

#### Yeast data

The yeast dataset used in the present work was that described in [15], and is available to the public [22]. This dataset is composed of expression data for 6200 yeast genes measured at 17 time points in the time period of 0–160 min. We used a same selection of 2945 genes, as described in [8].

### Clustering algorithm

Hard clustering methods allocate each gene to a single cluster only. These methods perform well if the boundaries between clusters are well defined. In real situations, however, clusters may overlap. Such overlaps are especially likely in gene expression data, because genes may have characteristics typical of more than one gene cluster. The fuzzy clustering method connects each gene to all clusters by way of an indicator vector. The elements of the indicator vector correspond to the degrees of membership of the gene to the various clusters, where the membership has a value between 0 and 1. A membership of close to 1 indicates that the gene has a strong association to the cluster, whereas a membership close to 0 indicates a weak association. The goal of the fuzzy clustering method is to evolve a partition matrix **W**(**X**) of a given dataset, **X **= {**x**_1_, **x**_2_,...,**x**_n_}, to find *c *clusters, 2 ≤ *c *≤ *n*, and a *c*-partition of **X **exhibiting categorically homogeneous subsets. Here, **x**_*i *_represents the normalized, expression level of gene *i*, in any array. The partition matrix **W **= (*w*_*ik*_) is of size *n *× *c*, where *w*_*ik *_is the membership value of gene *i *(*i *= 1,...,*n*) for the cluster *k *(*k *= 1,...*c*).

#### Fuzzy c-means clustering

Fuzzy c-means clustering can be represented as follows [9]:

minimize Jfcm(W,V)=∑i=1n∑k=1c(wik)m||xi−vk||2,
 MathType@MTEF@5@5@+=feaafiart1ev1aaatCvAUfKttLearuWrP9MDH5MBPbIqV92AaeXatLxBI9gBaebbnrfifHhDYfgasaacH8akY=wiFfYdH8Gipec8Eeeu0xXdbba9frFj0=OqFfea0dXdd9vqai=hGuQ8kuc9pgc9s8qqaq=dirpe0xb9q8qiLsFr0=vr0=vr0dc8meaabaqaciaacaGaaeqabaqabeGadaaakeaaieGacqWFTbqBcqWFPbqAcqWFUbGBcqWGPbqAcqWGTbqBcqWGPbqAcqWG6bGEcqWGLbqzcqqGGaaicqWGkbGsdaWgaaWcbaGaemOzayMaem4yamMaemyBa0gabeaakiabcIcaOiabdEfaxjabcYcaSiabdAfawjabcMcaPiabg2da9maaqahabaWaaabCaeaacqGGOaakcqWG3bWDdaWgaaWcbaGaemyAaKMaem4AaSgabeaakiabcMcaPmaaCaaaleqabaGaemyBa0gaaaqaaiabdUgaRjabg2da9iabigdaXaqaaiabdogaJbqdcqGHris5aaWcbaGaemyAaKMaeyypa0JaeGymaedabaGaemOBa4ganiabggHiLdGccqGG8baFcqGG8baFieqacqGF4baEdaWgaaWcbaGaemyAaKgabeaakiabgkHiTiab+zha2naaBaaaleaacqWGRbWAaeqaaOGaeiiFaWNaeiiFaW3aaWbaaSqabeaacqaIYaGmaaGccqGGSaalaaa@686C@

where *J*_*m*_(*W*,*V*) represents the objective function defining the quality of the result obtained for prototypes V and membership W, and *m *is the degree of fuzzification in the clustering. The membership degrees *w*_*ik *_are defined such that 0 ≤ *w*_*ik *_≤ 1, under the constraint of ∑k=1cwik=1
 MathType@MTEF@5@5@+=feaafiart1ev1aaatCvAUfKttLearuWrP9MDH5MBPbIqV92AaeXatLxBI9gBaebbnrfifHhDYfgasaacH8akY=wiFfYdH8Gipec8Eeeu0xXdbba9frFj0=OqFfea0dXdd9vqai=hGuQ8kuc9pgc9s8qqaq=dirpe0xb9q8qiLsFr0=vr0=vr0dc8meaabaqaciaacaGaaeqabaqabeGadaaakeaadaaeWbqaaiabdEha3naaBaaaleaacqWGPbqAcqWGRbWAaeqaaaqaaiabdUgaRjabg2da9iabigdaXaqaaiabdogaJbqdcqGHris5aOGaeyypa0JaeGymaedaaa@39E4@ for *i *= 1,...,*n*. **V **= (**v**_k_) is the cluster center or prototype, and ||**x**_*i *_- **v**_*k*_||^2 ^is the Euclidean distance between gene *i *and the prototype of cluster *k*. The advantages of the FCM approach are that it is unsupervized and always converges. The shortcomings of this approach, however, are that it searches only for the clustering solution closest to the starting center, and tends to give low degrees of membership for noisy points.

### Data normalization

It is important to remove from microarray data variations due to non-biological factors [18]. This process, known as normalization, is important for obtaining reliable data for subsequent analysis.

#### Common scale and location transformation

One of the most commonly utilized normalization approaches is the scale and location transformation, whereby all data are normalized such that every gene has a mean expression value of 0 and a standard deviation of 1 across the time point (we called Z-method), another method is based on the rank order of objects. In this method, all data are normalized by subtracting off the median followed by dividing by the quartile range [20] (we called R-method).

#### Lowess-normalization

One of the most commonly used nonlinear correction methods is locally weighted scatter plot smoothing (Lowess), which was first applied to microarray data by Yang et al. [23]. The main idea of Lowess is to utilize a locally weighted polynomial regression of the intensity scatter plot to obtain the calibration factor. Compared to other methods, the Lowess method is known to be robust across a wider range of types of datasets.

### Validation of clusters

#### Evaluation of final clusters

We used the adjusted Rand index to evaluate the final clusters [24]. This measure computes the average value of agreement between two partitions. Given a set of *N *genes, *D *= {*o*_1_, *o*_2_,...*o*_*N*_}, suppose **U **= {*u*_1_,*u*_2_,...,*u*_*R*_} and **V **= {*v*_1_,*v*_2_,...,*v*_*C*_} represent two different partitions of the genes in *D*. Here, for 1 ≤ *i *≠ *i*' ≤ *R *and 1 ≤ *j *≠ *j*' ≤ *C*, ∪i=1Rui=∪j=1Cvj=D
 MathType@MTEF@5@5@+=feaafiart1ev1aaatCvAUfKttLearuWrP9MDH5MBPbIqV92AaeXatLxBI9gBaebbnrfifHhDYfgasaacH8akY=wiFfYdH8Gipec8Eeeu0xXdbba9frFj0=OqFfea0dXdd9vqai=hGuQ8kuc9pgc9s8qqaq=dirpe0xb9q8qiLsFr0=vr0=vr0dc8meaabaqaciaacaGaaeqabaqabeGadaaakeaadaWeWaqaaiabdwha1naaBaaaleaacqWGPbqAaeqaaaqaaiabdMgaPjabg2da9iabigdaXaqaaiabdkfasbqdcqWIQisvaOGaeyypa0ZaambmaeaacqWG2bGDdaWgaaWcbaGaemOAaOgabeaaaeaacqWGQbGAcqGH9aqpcqaIXaqmaeaacqWGdbWqa0GaeSOkIufakiabg2da9iabdseaebaa@41C5@ and *N*_*ij *_is the number of genes that are in both classes *u*_*i *_and *v*_*j*_, and *N*_*i. *_and *N*_*.j *_are the numbers of genes in classes *u*_*i *_and *v*_*j *_respectively. The adjusted Rand index is as follows [24,25]:

adjRand=∑ij NijC2−⌊∑i Ni.C2∑j N.jC2⌋/NC2(1/2)[∑i Ni.C2+∑j N.jC2]−[∑i Ni.C2∑j N.jC2]/NC2
 MathType@MTEF@5@5@+=feaafiart1ev1aaatCvAUfKttLearuWrP9MDH5MBPbIqV92AaeXatLxBI9gBaebbnrfifHhDYfgasaacH8akY=wiFfYdH8Gipec8Eeeu0xXdbba9frFj0=OqFfea0dXdd9vqai=hGuQ8kuc9pgc9s8qqaq=dirpe0xb9q8qiLsFr0=vr0=vr0dc8meaabaqaciaacaGaaeqabaqabeGadaaakeaacqWGHbqycqWGKbazcqWGQbGAcqWGsbGucqWGHbqycqWGUbGBcqWGKbazcqGH9aqpdaWcaaqaamaaqababaGaem4qam0aaSbaaSqaaiabikdaYaqabaaabaGaemyAaKMaemOAaOMaeeiiaaIaemOta40aaSbaaWqaaiabdMgaPjabdQgaQbqabaaaleqaniabggHiLdGccqGHsisldaGbdaqaamaaqababaGaem4qam0aaSbaaSqaaiabikdaYaqabaaabaGaemyAaKMaeeiiaaIaemOta40aaSbaaWqaaiabdMgaPjabc6caUaqabaaaleqaniabggHiLdGcdaaeqaqaaiabdoeadnaaBaaaleaacqaIYaGmaeqaaaqaaiabdQgaQjabbccaGiabd6eaonaaBaaameaacqGGUaGlcqWGQbGAaeqaaaWcbeqdcqGHris5aaGccaGLWJVaay5+4dGaei4la8YaaSbaaSqaaiabd6eaobqabaGccqWGdbWqdaWgaaWcbaGaeGOmaidabeaaaOqaaiabcIcaOiabigdaXiabc+caViabikdaYiabcMcaPmaadmaabaWaaabeaeaacqWGdbWqdaWgaaWcbaGaeGOmaidabeaaaeaacqWGPbqAcqqGGaaicqWGobGtdaWgaaadbaGaemyAaKMaeiOla4cabeaaaSqab0GaeyyeIuoakiabgUcaRmaaqababaGaem4qam0aaSbaaSqaaiabikdaYaqabaaabaGaemOAaOMaeeiiaaIaemOta40aaSbaaWqaaiabc6caUiabdQgaQbqabaaaleqaniabggHiLdaakiaawUfacaGLDbaacqGHsisldaWadaqaamaaqababaGaem4qam0aaSbaaSqaaiabikdaYaqabaaabaGaemyAaKMaeeiiaaIaemOta40aaSbaaWqaaiabdMgaPjabc6caUaqabaaaleqaniabggHiLdGcdaaeqaqaaiabdoeadnaaBaaaleaacqaIYaGmaeqaaaqaaiabdQgaQjabbccaGiabd6eaonaaBaaameaacqGGUaGlcqWGQbGAaeqaaaWcbeqdcqGHris5aaGccaGLBbGaayzxaaGaei4la8YaaSbaaSqaaiabd6eaobqabaGccqWGdbWqdaWgaaWcbaGaeGOmaidabeaaaaaaaa@967E@

We would expect a high value of the adjusted Rand index to indicate good clustering.

#### Determination of the number of clusters

We used the silhouette index [7] to estimate the number of clusters [24]. The silhouette width for the *i-*th gene in cluster *j *is defined as s(i)=bj(i)−aj(i)max⁡{aj(i),bj(i)}
 MathType@MTEF@5@5@+=feaafiart1ev1aaatCvAUfKttLearuWrP9MDH5MBPbIqV92AaeXatLxBI9gBaebbnrfifHhDYfgasaacH8akY=wiFfYdH8Gipec8Eeeu0xXdbba9frFj0=OqFfea0dXdd9vqai=hGuQ8kuc9pgc9s8qqaq=dirpe0xb9q8qiLsFr0=vr0=vr0dc8meaabaqaciaacaGaaeqabaqabeGadaaakeaacqWGZbWCcqGGOaakcqWGPbqAcqGGPaqkcqGH9aqpdaWcaaqaaiabdkgaInaaBaaaleaacqWGQbGAaeqaaOGaeiikaGIaemyAaKMaeiykaKIaeyOeI0Iaemyyae2aaSbaaSqaaiabdQgaQbqabaGccqGGOaakcqWGPbqAcqGGPaqkaeaacyGGTbqBcqGGHbqycqGG4baEcqGG7bWEcqWGHbqydaWgaaWcbaGaemOAaOgabeaakiabcIcaOiabdMgaPjabcMcaPiabcYcaSiabdkgaInaaBaaaleaacqWGQbGAaeqaaOGaeiikaGIaemyAaKMaeiykaKIaeiyFa0haaaaa@52E1@, where *a*_*j*_(*i*) is the average distance between the *i*-th gene and all of the genes in the *j*-th cluster, and *b*_*j*_(*i*) is the smallest average distance between the *i*-th gene and all of the genes in the *l*-th cluster (1 ≤ *j*,*l *≤ *k*,*j *≠ *l*). Thus, for a given cluster, a cluster silhouette value of silj=∑i=1Njs(i)/Nj
 MathType@MTEF@5@5@+=feaafiart1ev1aaatCvAUfKttLearuWrP9MDH5MBPbIqV92AaeXatLxBI9gBaebbnrfifHhDYfgasaacH8akY=wiFfYdH8Gipec8Eeeu0xXdbba9frFj0=OqFfea0dXdd9vqai=hGuQ8kuc9pgc9s8qqaq=dirpe0xb9q8qiLsFr0=vr0=vr0dc8meaabaqaciaacaGaaeqabaqabeGadaaakeaacqWGZbWCcqWGPbqAcqWGSbaBdaWgaaWcbaGaemOAaOgabeaakiabg2da9maaqahabaGaem4CamNaeiikaGIaemyAaKMaeiykaKIaei4la8IaemOta40aaSbaaSqaaiabdQgaQbqabaaabaGaemyAaKMaeyypa0JaeGymaedabaGaemOta40aaSbaaWqaaiabdQgaQbqabaaaniabggHiLdaaaa@43B7@ characterizes the heterogeneity and isolation properties of the cluster, where *N*_*j *_is the number of genes in the *j*-th cluster. Thus the number of clusters that maximizes *ave sil *is taken as the optimal number of clusters, *c*.

## Authors' contributions

S.Y. Kim designed and carried out the comparative study, wrote the code, and drafted the manuscript. J.W. Lee brought up the biological problem and provided discussion on the methodology. J.S. Bae participated in the design of the study and coordination. All authors read and approved the final manuscript.

## Figures and Tables

**Table 1:** Parameters used for the FCM clustering of the real datasets. Comparison of fuzzification, *m*, and number of clusters, *c*, according to the different normalization methods (values in parentheses are the numbers of clusters for the normalized data). *N *and *p *indicate the numbers of genes and arrays for the dataset used in the analysis, respectively. In particular, selection of *m *depends on the normalization method applied to the dataset.

			Normalization method		
			
Dataset	*N*	*p*	Z (c)	R (c)	Lowess (c)
Serum	517	13	1.25 (10)	1.30 (9)	1.30 (11)
Sporulation	522	16	1.63 (11)	1.75 (11)	1.66 (12)
Yeast	2945	7	1.16 (10)	1.28 (9)	1.66 (13)

**Table 2:** Validation of FCM clustering for simulated data by normalization. Adjusted Rand indices for clusters by applying the FCM method to three simulated datasets normalized using three methods, where the fuzzification (*m*) value used was either the optimal value or *m *= 2.

Data\method	When *m *is optimal			When *m *is fixed to 2		
	
	Z	R	Lowess	Z	R	Lowess
SD1/450	0.47	0.44	0.82	0.49	0.40	0.81
SD2/450	0.43	0.38	0.70	0.49	0.41	0.77
SD3/180	0.12	0.10	0.60	0.15	0.14	0.58

**Table 3:** Comparison results for clusters obtained using the FCM method and three hard clustering methods for the three simulated datasets. Adjusted Rand indices for clusters obtained using the FCM method and the hard clustering methods PAM, K-means and Hierarchical clustering for the three simulated datasets.

Data	FCM	PAM	K-means	Hierarchical
SD1/450	0.85	0.98	0.55	1.00
SD2/450	0.70	0.85	0.71	0.83
SD3/180	0.69	0.63	0.59	0.61
